# Case report: binaural beats music assessment experiment

**DOI:** 10.3389/fnhum.2023.1138650

**Published:** 2023-05-05

**Authors:** Elizabeth Krasnoff, Gaétan Chevalier

**Affiliations:** ^1^California Institute for Human Science, Encinitas, CA, United States; ^2^Department of Family Medicine and Public Health, University of California, San Diego, La Jolla, CA, United States

**Keywords:** binaural beats, neural rhythms, auditory illusion, frequency following, audio beats, neural response

## Abstract

We recruited subjects with the focus on people who were stressed and needed a break to experience relaxation. The study used inaudible binaural beats (BB) to measure the ability of BB to induce a relaxed state. We found through measuring brain wave activity that in fact BB seem to objectively induce a state of relaxation. We were able to see this across several scores, F3/F4 Alpha Assessment and CZ Theta Beta, calculated from EEG readings, that indicated an increase in positive outlook and a relaxing brain, respectively, and scalp topography maps. Most subjects also showed an improvement in Menlascan measurements of microcirculation or cardiovascular score, although the Menlascan scores and Big Five character assessment results were less conclusive. BB seem to have profound effects on the physiology of subjects and since the beats were not audible, these effects could not be attributed to the placebo effect. These results are encouraging in terms of developing musical products incorporating BB to affect human neural rhythms and corollary states of consciousness and warrant further research with more subjects and different frequencies of BB and different music tracks.

## Goal

The aim of this case study is to assess the effects of adding Binaural Beats (BB) to brown noise and to music on the brain and physiology of four study participants.

## Introduction

Binaural beats (BB) are a perceptual beat frequency created by the brain in response to the differences between two audio beats captured by both ears (Avan et al., 2015). Two brainstem circuits compare the sound that is captured by each ear. The brain combines information about sounds received by the ears and produces an auditory illusion, a frequency following response that operates by stimulating neuronal phase locking (Avan et al., 2015). The brain can only identify the illusion if two receivers detect the tones (Avan et al., 2015).

Binaural beats are currently the focus of scientific interest after first being described by Dove and Moser (1839); Royal Academy of Arts and Sciences (2022) and then defined by Thompson (1877) as a convergence of signals transmitted from the auditory nerves to the brain. They were reintroduced by physicist Oster (1973). Binaural cues are important for sound localization and separation of signals from noise (Fitzpatrick et al., 2009). The therapeutic goal is to send a specific frequency to guide the brain into the desired state. BB are hard to measure, offering only minimal frequency following responses (Oster, 1973). Researchers intending to prove auditory entrainment reported too weak a signal, and therefore no validation of BB (López-Caballero and Escera, 2017), however cross frequency or functional connectivity signaling was observed (Gao et al., 2014; Perez et al., 2020). Continued research begins to understand that BB do not create an auditory entrainment, and the weaker signal is a sign of something else. There are varying hypothesis regarding BB, which are essentially a transfer of energy and a delivery of electrical information into the brain, including that they are perhaps a resonance phenomenon such as stochastic resonance (Oster, 1973; Reedijk et al., 2015; Solcà et al., 2016). This is one of the first areas recommended for research. Stochastic resonance is a hypothesis because BB are also enhanced by noise, rather than masked (Oster, 1973), a phenomenon that is called the “cocktail party effect” (Bronkhorst, 2000).

The brain’s response to BB can be measured by EEG, but we do not yet know the full auditory signal pathway flow of BB. It is possible that BB are effective due to changes in brain oscillations that favor neurological gain and better response to auditory stimuli (Lakatos et al., 2008). The main theory is that BB travel a non-primary auditory pathway, the reticular activating system (RAS), and impact our states of consciousness through this gateway to conscious arousal in our brain (Atwater, 1997). Consciousness as such is defined in this field as an arousal and awareness of environment and self, which is achieved through action of the RAS on the brain stem and cerebral cortex (Daube et al., 2019; Paus, 2000; Zeman, 2001; Gosseries et al., 2011).

Binaural beats have been measured in the superior olivary nuclei, in the brains of cats (Rose et al., 1959). This is the second of four major destinations in the basic afferent (upstream) auditory signal flow, and the location of sound localization (Oster, 1973). There is a return downstream, or efferent auditory signal pathway, which is beyond the discussion of this paper (Kraus, 2021). We now have measured the presence of BB in the third efferent stop, the inferior colliculus (ICC), the principal auditory nucleus in the mid-brain. BB were used in an experiment, again in the brains of cats, to measure the neighboring neurons sensitivity to interaural time differences (ITD) in the ICC (Seshagiri and Delgutte, 2007). The characteristic phases of neighboring neurons showed a significant correlation, supporting the research that there is a segregation of inputs to the ICC from the lateral and medial superior olives. This sophisticated study is beginning to entangle the central auditory pathway of sound in general, including BB, and is the first evidence of the BB signal flow beyond the superior olives. Seshagiri and Delgutte (2007) also found that neighboring neurons showed similar preferences, suggesting that neurons are connected and work together to detect sounds. Spitzer and Semple (1998) tracked a binaural audio signal from the superior olivary complex (SOC) to the ICC, again verifying this signal path, and concluding that interaural phase-disparity (IPD), phasing information of the auditory object, is influenced and enriched at the ICC, possibly by temporal variation of IPD, allowing for higher up processing and enrichment of spatial location. This is a good model for a future parallel BB study. Beyond these two locations, BB had been measured at the final stop of the afferent auditory pathway, the auditory cortex (Pratt et al., 2010), and thought to be processed similarly to acoustic sound. Ungan et al. (2019) addressed this in a groundbreaking study. Measuring BB using EEG can be difficult since BB occur so quickly that the tones can overlap and interfere with the recording. Ungan et al. (2019) slowed the frequency of BB to produce single-cycle BB’s of a 250 Hz tone. This produced a larger response in the brain compared to prior methods, which allowed for deeper and more accurate assessment of the signal flow. This method allowed us to see that the cortical processing of BB were different than regular auditory cortical processing as previously reported (Pratt et al., 2010).

The impact of BB on individuals has been the focus of numerous studies. BB provide an opportunity to learn more about the workings of the auditory brain and binaural hearing, since the effects of the beats can be detected using EEG. The beats operate as brain-wave-like combinations that mimic the complex wave patterns of the brain (Atwater, 2001; Garcia-Argibay et al., 2019) and cause brain hemispheres to synchronize (Becher et al., 2015; Beauchene et al., 2016; Solcà et al., 2016; Perez et al., 2020). Becher et al. (2015) measured phase synchronization between brain channel pairs in response to hearing monaural and binaural beats. Brain synchronization increased in response to stimulation with monaural 10-Hz beats and binaural 5-Hz beats, suggesting that beat stimulation offered a non-invasive approach for the altering intracranial EEG characteristics. Specifically, Gamma and Theta BB frequencies can affect the brain cortex, causing increased functional connectivity including cross-frequency (Perez et al., 2020).

Systematic reviews have shown some positive outcomes of listening to BB, but more investigation is required. Aparecido-Kanzler et al. (2021) reported that most of the studies reviewed indicated BB effectiveness on brainwave states. A meta-analysis and systematic review by Basu and Banerjee (2022) on the effect of BB memory and attention reported an overall medium and significant effect size (*g* = 0.40), with conflicting results regarding theta and beta’s efficacy on memory and attention-related tasks. Results of a meta-analysis by Garcia-Argibay et al. (2019) based on 35 effect sizes showed an overall medium, significant, consistent effect size (*g* = 0.45). Duration of exposure was significant, showing indicating that longer periods of more than 9 min were advisable to ensure maximum effectiveness. Research using magnetoencephalographic (MEG) instruments showed responses to BB in the cerebral cortex (Karino et al., 2006).

A study of daytime sleep as activated by the parasympathetic function of the autonomic nervous system showed that BB supported the function (Bakaeva et al., 2022). Dabiri et al. (2022) found that delta frequency BB improved sleep and mood disorders. Jirakittayakorn and Wongsawat (2017) reported that increased theta brainwave activity throughout the brain cortex in response to BB resembled a meditative state (Jirakittayakorn and Wongsawat, 2017). Binaural beats can be used to enhance mental focus. Axelsen et al. (2020) investigated whether two types of interventions, mindfulness and Beta BB with music, could reduce mental fatigue and support cognitive focus. Results showed that the BB with music group and the experienced mindfulness group were least affected by mental fatigue.

The beats have been used to relieve anxiety and pain in medical settings. Sung et al. (2017) used familiar music with BB to cause immediate physical effects indicating relaxation and reduced depression. Opartpunyasarn et al. (2022) examined the effect of BB on anxiety in patients undergoing fiberoptic bronchoscopy. The experimental group reported reduced anxiety after listening to BB. Wiwatwongwana et al. (2016) investigated the use of BB to relieve anxiety. Patients in the BB group showed significant reductions in blood pressure and anxiety. Roshani et al. (2019) investigated the effect of BB with music on anxiety and pain of patients prior to eye surgery. Post-surgical anxiety and pain were significantly lower in the BB group than in the control group.

Perales et al. (2019) investigated the pain perception of BB users in a virtual reality environment using acoustic sound as the control group and BB as the experimental group. The aim was to induce relaxation and moderate pain perception. Results showed that BB worked better than acoustic stimulus. These research studies suggest that BB do have the power to change wave frequencies in the brain. Individuals can achieve deeper sleep (Dabiri et al., 2022), relaxation (Sung et al., 2017), reduced anxiety (Garcia-Argibay et al., 2019), pain relief (Perales et al., 2019), depression relief (Sung et al., 2017), creativity (Reedijk et al., 2013), or mental focus and concentration (Axelsen et al., 2020) by listening to a specific BB frequency.

Despite numerous reports of positive experimental outcomes (Sung et al., 2017; Perales et al., 2019; Axelsen et al., 2020; Dabiri et al., 2022) the results of some studies on BB have been inconclusive. Jirakittayakorn and Wongsawat (2017) attributed this inconsistency to variation in the factors such as beat frequency, carrier tone frequency, exposure duration and recording. As concerns exposure duration, a meta-analysis of efficacy of BB for cognition, anxiety, and pain perception (Garcia-Argibay et al., 2019) showed that a minimum duration of 8–9 min was necessary for an effect. López-Caballero and Escera (2017) reported inconclusive results for BB in brainwave entrainment. Lovati et al. (2019) also reported inconclusive findings regarding effects of BB for relief of headache, anxiety, depression and sleep problems. Solcà et al. (2016) found that while 4 and 10 Hz BB selectively synchronized the brain hemispheres, no behavioral effects were found. Munro and Searchfield (2019) found that the addition of Alpha (8 Hz) BB to an ocean sound produced no significant benefits for tinnitus sufferers. This relatively new research field is still determining its parameters.

Individual differences in brain chemistry can lead to conflicting results regarding the effectiveness of BB. Reedijk et al. (2013) aimed to investigate whether BB affected creative performance and whether effects were mediated by the individual dopamine level in response to BB of Alpha and Gamma frequencies. Results showed that individuals with low EBRs (low dopamine levels) benefitted on creative tasks in response to Alpha BB, while there was no effect on creative task performance by individuals with high EBRs (high dopamine levels); some showed creative task performance was impaired by Alpha and Gamma BB. A follow up study by Reedijk et al. (2015) investigated the effect of high-frequency.

Binaural beats on attentional control during an attentional eye blink task. The BB eliminated the eye blinks, but only in individuals with low EBR corresponding to low dopamine levels. The results of these two studies suggests that the way in which BB affect cognitive performance depends on individual differences in cognitive-control factors such as dopamine. This is an important factor for future research, however, it was beyond the scope of this small pilot study, requiring more time, more participants and more funding. Some investigations also reported gender differences concerning binaural-beat perception (Oster, 1973) and alterations in auditory perception during the menstrual cycle (Tobias, 1965; Haggard and Gaston, 1978), as well as in older persons (Grose and Mamo, 2012).

## Case description

The pilot study flyer advertised for individuals who were looking to take a break from their stress and experience profound relaxation. Recruitment was done by random sampling by posting flyers, electronic marketing, and by snowball sampling. All participants read and signed a standard Informed Consent form prior to participation in the study. Written informed consent was obtained from the individuals for the publication of any potentially identifiable images or data. Exclusionary criteria included having a serious illness of inability to abstain from medication or stimulants on the days of the study. Four adults were randomly selected and participated twice on two consecutive days, two male participants in the first week, and two female participants in the second week, 1 week apart. A total of four auditory stimulations were administered during their two sessions, each session (Part 1 and Part 2) having two stimulations. The room was kept dark and quiet, and the participants were read a 1-min relaxation script written by the sponsor prior to each session. The same relaxation script was used for each session.

The 4-day study began 31 October, 2021. Recruitment began 1 week earlier, by flyer and email. All four subjects were tested at exactly the same times on all 4 days, to control for circadian rhythms, which may impact response to BB (Webb and Dube, 1981; Rossi, 1986; Shannahoff-Khalsa, 1991; Atwater, 2001). The following is the schedule:

Week 1 Day 1: Subject 1 -9a.m., Subject 2– 1p.m.

     Day 2: Subject 2- 9a.m., Subject 1–1p.m.

Week 2 Day 3: Subject 3- 9a.m., Subject 4–1p.m.

     Day 4: Subject 4- 9a.m., Subject 3–1p.m.

Each participant listened to the control track first and then the BB enhanced track second, so that we could clearly measure the difference between the Control Condition and the BB Condition. Subject 1 and 3 began Day 1 at 9a.m., with Part 1 (Conditions 1 and 2), and subjects 2 and 4 began Day 1 at 1p.m., with Part 2 (Conditions 3 and 4). This allowed everyone to listen to their four musical tracks at the same time of day. The two sequences were: Part 1: Condition 1 (brown noise only) and Condition 2 (brown noise plus binaural beats) and Part 2: Condition 3 (brown noise plus music) and Condition 4 (brown noise plus music plus binaural beats).

The study Sponsor provided the four auditory stimulation files, which were unidentified except for numbers 1 to 4, to provide a double-blind condition. The double-blind condition was met by keeping the track identities secret: neither the participants, nor the lab assistant, knew which track contained which condition. The tracks were presented to the lab only named 1–4 so no one knew what those numbers represented. The control condition was met by having each audio condition created both with and without the BB, and presenting the non-BB condition first, so that the difference could be tracked and measured.

The study was conducted at PsyTek Labs, a licensed clinical and public health research lab, in collaboration with the California Institute for Human Science (CIHS), by principal investigator Gaétan Chevalier, Ph.D., who is the Research Director.

## Participants

The four participants were all in good health according to the demographic data they provided, any minor health conditions and medications are noted below.

SUBJECT 1 is a 52 yo male (DOB 7/31/1969) dealing with high blood pressure and a stress level of 5 on a Likert scale of 1 to 10. Subject 1 experiences stress due to money and career ambition, which affects his emotional health, and he is looking for help regulating sleep and lowering his stress levels. Exercise and meditation works for this individual.

SUBJECT 2 is a 46 yo male (DOB 9/9/72) experiencing little stress in his life, a 3 on a Likert scale of 1–10, and reports no other physical, mental or sleep issues, just managing high blood pressure with the medication Norvasc. He is looking to enjoy the retirement phase of his life. He is just beginning his journey into self care.

SUBJECT 3 is a 33 yo female (DOB 02/04/1988) who has a stress level of 5 on a Likert scale of 1 to 10. Subject 3 experiences stress due to work and relationships, which affects her emotional and physical health. She would like to achieve more relaxation, lower her stress, and sleep better. Her self-care program involves relaxation music which helps her to fall asleep at night immediately. She reports no significant physical or mental issues and is taking no medications.

SUBJECT 4 is a 52 yo female (DOB 3/22/1969) who has predominantly anxiety at a level of 3 on a Likert scale of 1 to 10. Subject 4 experiences menopausal symptoms such as anxiety and poor sleep, for which she takes Bijuva. Her anxiety is amplified from work and poor organization/planning ahead skills, and she would like to use BB to experience greater relaxation. She has tried over the counter sleep aids.

## Instruments

### Binaural beats

Headphones are required for using binaural beats. The study Sponsor supplied the headphones. All participants used the same brand and serial number of headphones (Blue Lola Sealed Over-Ear) and the same auditory volume (40 dB) for all audio conditions. The musical track was composed in the ambient, relaxation genre using piano music and synthesizer pads at a tempo of 60 bpm. Binaural Beats were added in Pro tools, a professional audio production software. The musical track was titled Relax 1. The BB added were Theta at 4 Hz (4 cycles per second) and Alpha at 8 Hz (8 cyles per second). These frequencies were chosen for their correlation to deep, inward, yet awake meditative states (Shapero and Prager, 2020). The carrier frequency selected was 440 Hz. The exact formula was 438 Hz in the left ear and 442 Hz in the right ear for Theta, and 436 Hz in the left ear and 444 Hz in the right ear for Alpha. The carrier frequency of 440 Hz was selected due to the research that frequencies in this 400 range are the most effective due to the size of the human skull (Oster, 1973; Atwater, 2001). The sponsor chooses to disclose all formulas for the enrichment of the research community as a whole, and invites all researchers to do the same. The sponsor has previously made all BB dissertation research available open source to this community.

A combination Alpha-Theta frequency was chosen due to the findings of Garcia-Argibay et al. (2019), that mixed frequencies were more effective. This was also the conclusion of pioneer researcher Atwater (2001). The sponsor chose to make the BB (and brown noise) inaudible after reading research showing that BB still produce effects even when one of the carrier frequencies is below hearing (Oster, 1973). The added benefit of inaudible BB was the removal of the placebo effect, given that the BB were inaudible and no one knew when they were present. All tracks were the same length of 10 min, due to the research showing that a minimum of 8 min is needed for the brain to resonate to the offered frequencies (Garcia-Argibay et al., 2019). Brown noise contains all frequencies, like white noise, but emphasizes the lower frequencies, and de-emphasizes the higher frequencies (Blum and Jamet, 2022). The brown noise was chosen due to research showing that pink noise is an effective binaural beats carrier (Garcia-Argibay et al., 2019). The sponsor selected brown noise instead due to personal preference, it is much easier and more pleasant to listen to 10 min of brown noise rather than pink noise, and brown noise is a comparable full spectrum sound. The sponsor hypothesized that adding an unchanging brown noise carrier tone under the musical track, which has variations, would more effectively carry the BB. The numerical data extracted from the EEG analysis supported this outcome. Due to time limitations, the condition of music and BB only without the brown noise was not tested. Further research including this condition is recommended.

### WAVi P300

The instrument used to measure the functioning of the brain was the brain scan platform WAVi P300 Brain Mapping System. WAVi is a non-invasive, HIPAA-compliant brain measurement platform which provides data about brain function using EEG technology to measure brain activity in response to a stimulus (Grover et al., 2017; Tarrant et al., 2019; Joffe et al., 2021; Oakley et al., 2021; Mortazavi et al., 2023). WAVi can be considered as research-friendly since EEG with audio P300 has been used in health screening exams for research conducted by hospitals and clinics for clinical evaluations (Joffe et al., 2021). The P300 wave is an electrical response of the brain that shows the brain’s response to stimuli as well as how quickly or easily the response occurs (Tarrant et al., 2019). P300 is considered to be an endogenous potential, as its occurrence links not to the physical attributes of a stimulus, but to a person’s reaction to it. More specifically, the P300 is thought to reflect processes involved in stimulus evaluation or categorization. The EEG signals are processed, cleaned and the P300 extracted by the WAVi software.

## Menlascan

Menlascan (Menla Technologies, Independence, Missouri) provided measurements on the cardiovascular system in response to the auditory stimulus, which refers to the heart (cardio) and blood vessels (vascular). This system distributes blood to all parts of the body and is governed by the autonomic nervous system (ANS). The ANS is a component of the peripheral nervous system that regulates involuntary physiologic processes including heart rate, blood pressure, respiration, digestion, and sexual arousal (Waxenbaum et al., 2022). Microcirculation is blood flow through the smallest vessels of the cardiovascular system. The most important results here are the improvement of cardiovascular and microcirculation scores as the ANS is prone to rapid changes with emotions.

## Big Five Inventory

The theory of five basic personality traits (Big Five) was developed by Fiske (1995). The five basic personality traits described by the theory include extraversion/extroversion, agreeableness, openness, conscientiousness, and neuroticism. Evidence of this theory has been reported by researchers including Kachur et al. (2020), and in meta-analyses by Buecker et al. (2020), Plessen et al. (2020), and Mammadov (2022).

## BMIS assessments

The Brief Mood Introspection Scale (BMIS) is an open-source mood scale based on 16 mood-adjectives such as “Are you” “happy?” Mayer and Gaschke (1988). The scale yields measures of moods including pleasant-unpleasant mood, arousal-calm mood, as well as scores for positive-tired and negative-calm mood (Mayer and Gaschke, 1988). The scale has been validated in numerous studies including Nugraha et al. (2020), Aldrich et al. (2021), and Flexer et al. (2021). In order to evaluate any possible correlation between subjective assessment and quantitatively gathered data, participants filled out a BMIS form after each condition. Seven of the eight forms were completed. In addition to present time mood assessments, an overall mood assessment on a scale of 1 to 10 was requested.

## Results

### Scalp topography

Scalp topography maps are provided for each participant. These scalp topography maps were computed from the 19 EEG channels recorded by the brain mapping system (frontal part of brain at the top and back of head at the bottom of each circle). These maps show the amplitude of the P300 recording for each channel (in microvolts or uV) using a color-coding scale presented on the right of each topographical map (Minimum value uV, blue; Maximum value 7 uV, red). Red colors indicate excitement and activity, and blue colors indicate calm and less activity. The scalp topography maps are a visual representation of the activity of the different regions of the brain on this 1 to 7 scale measurement of the amplitude of the P300 response. The number 7 corresponds to 7 microvolts (the color red) meaning the highest brain response (or brain activation) at all frequencies combined. The number 1 (and the color blue) is the lowest amplitude and the lowest or calmest brain activation at all frequencies combined. Due to size limitations, we have not included the amplitude or coherence data for each frequency band. We did use the two following assessment scores from the WAVi P300 data.

### Theta beta assessment scores

Theta and Beta frequency bands are affected by cortical arousal and can give insight into how the brain functions. Lowering of CZ is the clearest indication that the brain has calmed down. For this measurement, descending numbers will indicate a positive effect.

### State F3/F4 alpha assessment score

Calculating F3/F4 relative power ratio in the Alpha band allows us to understand if the brain is processing information in a positive way. An increase in the ratio indicates a positive processing mode, while a decrease in the ratio indicates a more negative processing mode. For this measurement, ascending numbers will indicate a positive effect.

### Big Five Inventory

We administered the Big Five Inventory (BFI) character assessment to determine if the subjects were introverted or extroverted (see graphic). Research shows that BB are more effective on extroverted personalities (Chaieb et al., 2015). Subject 1, Male, 52, and subject 2, Male 46, both show a tendency to extravert, with strong tendencies toward Agreeableness, Conscientiousness and Openness and low tendencies toward Neuroticism. Subject 3, Female, 33, is more of an introvert than an extrovert. She is quite balanced regarding the dimensions of Agreeableness, Conscientiousness, and neuroticism with a strong tendency toward Openness. Female, 52. Subject 4 shows a slight tendency to be an extravert and to be open, with strong tendencies toward Agreeableness and Conscientiousness. She is quite average in the Neuroticism dimension. Given the research we would expect to see that the introverted subject is less affected than the extroverted subjects, but the EEG and cardiovascular measurements showed no observable difference in effect. There is not enough data to come to a full conclusion, but the sponsor is wanting to model that this data should be included in every binaural beats BB study given the information that introversion and extroversion are pertinent factors. A similar study with a greater number of participants is suggested. There are quite a few studies in theis a growing field of personality assessment and music preferences, indicating that these factors are of importance (Rentfrow et al., 2011; Anderson et al., 2021). Research shows that BB are more effective on extroverted personalities (Chaieb et al., 2015).

### Case results

The combined three WAVi P300 measurements for each study participant are as follows. The Menlascan results are summarized at the end.

## Subject 1

### State CZ theta beta assessment scores

Subject one began with a control score of 2.1, which lowered dramatically to 0.8 when BB were introduced to the brown noise. In the second session, the number lowered dramatically again from 2.7 to 1.1 when BB were introduced to the music.

### State F3/F4 alpha assessment score

Subject 1 experienced the opposite of the expected outcome, instead showing a lowering of numbers from 0.7 to 0.2 when BB were added to the brown noise, indicating a more negative processing mode rather than a more positive mode. However, it correlated with his personal assessment, in which Subject 1 reported the brown noise as intolerable, saying: “Felt a “white noise” calm almost “ocean” but over time it became less calming and almost causing mild discomfort like if I was trapped in a loud “machine” room. When the headset was removed it was relief.” By contrast, when BB were added to the music in session 2, the expected positive increase from 0.7 to 1.5 was achieved.

### Scalp topography

**FIGURE 1:**
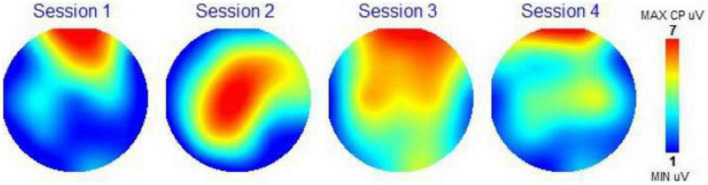
Scalp topography map subject 1.

Figure 1 shows scalp topography of the brain response for each session for Subject 1. In Session 1, we see that his otherwise calm brain became more attentive in the frontal area after brown noise, indicating thinking, but adding the BB in Session 2 moved the activity to the middle of the brain (location of the CZ point), indicating psychomotor or sensory processing. Condition 3 of music only created a very active brain, especially in the frontal areas. Condition 4 with the BB calmed down all but the frontal brain, a possible indication of the positive effects of BB.

## Subject 2

### State CZ theta beta assessment scores

Subject 2 began with a control score of 2.3, which lowered dramatically to 1.5 when BB were introduced to the brown noise. In the second session, the number lowered again from 2.6 to 2.5. When BB were introduced to the music.

### State F3/F4 alpha assessment score

Subject 2 began with a control score of 0.5 which expectedly rose to 0.9 when BB were added to the brown noise. When BB were added to the music in session 2, again the expected positive increase from 1.0 to 1.1 was achieved.

### Scalp topography

**FIGURE 2:**
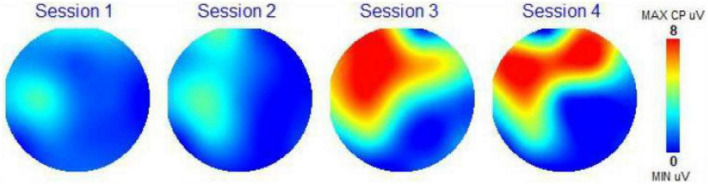
Scalp topography map subject 2.

Figure 2 shows scalp topography for each session for Subject 2. The brain of Subject 2 became slightly more attentive after adding BB in Session 2 in the area of sensorimotor integration. There was not much overall activity in response to the first two brown noise Conditions. The music in Session 3 and 4 seems to have had a profound effect on the brain of this subject in the left and temporal areas. These brain regions correspond to attention, motor planning, working and verbal memory and sensorimotor integration. Adding the BB increased attention in the right frontal lobe area, related to motor planning and emotional expression. It is possible that adding the BB made the music more emotional for him.

## Subject 3

### State CZ theta beta assessment scores

Subject 3 began with a control score of 1.8, which lowered dramatically to 1.0 when BB were introduced to the brown noise. In the second session, the number lowered again from 1.4 to 1.3. When BB were introduced to the music.

### State F3/F4 alpha assessment score

Subject three began with a control score of 1.2 which rose to 1.4 when BB were added to the brown noise. When BB were added to the music in session 2, the expected positive increase from 0.4 to 0.9 was achieved.

### Scalp topography

**FIGURE 3:**
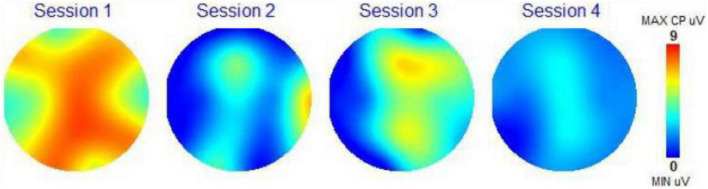
Scalp topography map subject 3.

Figure 3 shows scalp topography of the brain response for each session for Subject 3. It can be noted that after listening to Condition 1, her brain was aroused almost all over, indicating some level of agitation. After listening to Condition 2, the brown noise plus BB, her brain calmed down. After listening to Condition 3, the music track with brown noise, her brain became somewhat active, but not excessively, indicating that some memories were slightly activated (the top central yellow region) and that there was some cognitive processing (as shown by the bottom yellow region). After listening to Condition 4, the music track with brown noise and BB, her brain calmed down.

## Subject 4

### State CZ theta beta assessment scores

Subject 4 began with a control score of 1.0, which lowered to 0.9 when BB were introduced to the brown noise. In the second session, the number did not decrease, but rose from 0.9 to 1.5 when BB were introduced to the music. The unexpected increase may be attributed to processing of memories.

### State F3/F4 alpha assessment score

Subject 4 began with a control score of 1.1 which rose to 1.4 when BB were added to the brown noise. When BB were added to the music in session 2, again the expected positive increase from 1.1 to 1.8 was achieved.

### Scalp topography

**FIGURE 4:**
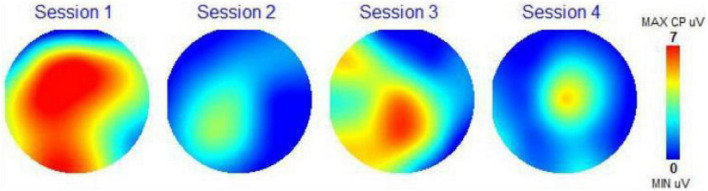
Scalp topography map subject 4.

Figure 4 shows scalp topography for Subject 4 for each session. It can be noted that after listening to Condition 1, brown noise, her brain was aroused almost all over, indicating some level of agitation. After listening to Condition 2, the brown noise plus BB, her brain relaxed quite a bit. After listening to Condition 3, the music track with brown noise, her brain became active in the central lower central region of the brain, a region performing cognitive processing. After listening to Condition 4, the music track with brown noise and BB, her brain calmed down further with the middle of the brain slightly active, a region performing sensorimotor integration.

### Menlascan

The Menlascan results (see Table with Menlascan Results TK) provided measurements on the cardiovascular system and microcirculation, the most important results here are the improvement of cardiovascular and microcirculation scores as the ANS is prone to rapid changes with emotions.

Subject 1 had the highest microcirculation score with Condition 2, brown noise and BB, and an increased cardiovascular score from Condition 4, music and BB (although just the control musical track increased cardiovascular as well).

For subject 2, there was no improvement with Condition 2, brown noise and BB. Condition 4, music and BB, increase both the cardiovascular and microcirculation score. See Figure 2 for image.

For subject 3, our introverted subject, we see the least effect, with both Condition 1, brown noise, and Condition 2, brown noise and BB, increasing only cardiovascular score, and Condition 4, music and BB providing only a mild cardiovascular improvement. See Figure 3 for image.

In subject 4, there is a mild improvement only in cardiovascular after Condition 2, brown noise and BB, Condition 3 and Condition 4. See Figure 4 for image.

## Discussion

### Summary by subject

In conclusion, Subject 1 became more attentive after Session 2 and Session 4, a possible indication of the positive effects of BB. Additionally, the brain of this subject became calmer after Session 4, a possible additional benefit of combining BB with music. Subject 1 had the highest microcirculation score with Session 2, brown noise and BB, and an increased cardiovascular score from Session 4, music and BB (although just the control musical track increased cardiovascular as well). These results agree with a personality that is conscientious and open (Supplementary material).

Subject 2 became more attentive after Session 2 and had a more positive mood while the music seems to have had a profound effect on this subject. Adding BB to brown noise and to music with brown noise seems to have helped this subject to be more present and have a more positive mode of processing information. The cardiovascular and microcirculation results were unremarkable. This subject overall has an excellent cardiovascular system which agrees with his personality being very stable. These results are consistent with the personality of this subject which is very stable emotionally with tendencies to agreeableness and openness.

After listening to the music track with brown noise, Subject 3’s brain became somewhat active, but not excessively, indicating that some memories were slightly activated. After listening to the music track with brown noise and BB, her brain calmed down. A notable change that occurred when comparing before and after listening to Condition 2, brown noise and BB, is a decrease in microcirculation score that is unexpected. Also unexpectedly, after listening to brown noise with BB, the cardiovascular score dropped as well. However, when Subject 3 came to the lab the next day (day 2) her microcirculation and cardiovascular scores had improved, possibly from the effects of the BB. After listening to Condition 3, music with brown noise, the only change is a decrease in microcirculation score. After listening to music plus brown noise with BB, her microcirculation score also decreased. Maybe she did not like the music? This subject is more of an introvert than an extrovert. Perhaps here we see the interaction of this quality, and the above results are related to this feature of her personality?

After listening to Condition 2, the brown noise plus BB, Subject 4’s brain relaxed. The same happened when listening to Condition 4, music plus brown noise and BB. The coherence results and the Auditory P300 show that even though she was relaxed, her brain was active and vigilant. Physical reaction time is normal and so is visual/psychomotor coordination. She has a positive processing brain which became even more so after adding BB. These results are consistent with a person that has a high level of conscientiousness.

After listening to Condition 2, the brown noise and BB, Subject 4’s brain relaxed quite a bit. The same happened when listening to Condition 4, music plus brown noise and BB. She has a positive processing brain (F3/F4 scores) which became even more so after adding BB. Subject 4’s Menlascan scores did not change much after listening to brown noise with the exception of the cardiovascular score going down, however, this score improves after listening to Condition 2, brown noise with BB. After listening to Condition 4, music plus brown noise with BB, both her microcirculation and cardiovascular scores improved. BB seems to be enhancing whatever this subject hears. Subject 4 has strong tendencies toward Agreeableness and Conscientiousness. Maybe she did not like brown noise alone, but she liked the music which mitigated the effect of the brown noise. Adding BB seems to have the effect of increasing this subject’s scores.

Based on these results, all four recipients responded positively to therapeutic relaxation from listening to BB. For the most part, they left the lab feeling better and more relaxed than when they arrived per our data, and their own BMIS self-assessment—especially after BB was added to relaxation music. Given their individual preferences and needs, the sponsor would recommend Alpha-Theta relaxation BB music as a form of meditation for Subject 1, 3 and 4. It is best to listen to the music 10 min a day, and headphones must be worn. Best times are during morning or evening meditation practice times, or in the afternoon for a rest and recharge, or integration of a busy day. Subject 1, 3, and 4 would benefit from Delta BB sleep tracks, best listened to before sleep. And subject 2 would benefit from an active meditation, such as an Alpha-Theta BB walking tracks. Subject 2 would also benefit from Alpha-Theta BB tracks for creativity, as he plans the second chapter of his life in retirement. Subject 3 would also find support for their menopausal lack of focus and disorganization with Beta BB focusing tracks. These recommendations essentially lay the framework for future studies with large sample sets of individuals, consistently using the BB tracks 10 min a day, Monday through Friday for a period of 6 weeks. A home measurement device to measure the brain would need to be employed, as well as self assessment questionairres. There is particularly the question of how menopausal women would respond to BB, given the hormonal component of the response to BB (Tobias, 1965).

The sponsor chose within-subject design to specifically test all independent control variables (noise, noise and BB, music, music, and BB) on all subjects, wanting to know if adding the BB into the exact same condition would provide any differences at all. As it happens it did. In order to make sure that each condition was cleanly measured, we gave enough time for participants to return to baseline conditions, in addition to having the part one and part two on two different days, but it is important to remember that the brain always changes and a new baseline had to be established for each experiment. Either way, four subjects are not enough to conduct statistical analysis or to do between subject comparison. What this study hopes to illustrate, is that it is important to begin to develop protocols for studying BB, so that larger studies can be conducted and their results can be compared.

With the scalp topography maps, we could visually see the impact of the four auditory conditions on the electrical activity of the brain, changing its activity in response to the control state, to a different activity picture. In most instances we are seeing a calmer brain when BB is added to the auditory condition. The CZ Theta Beta scores and F3/F4 Alpha scores presented a clear path toward observing the impact of the BB conditions on the specific frequency bands in the brain. Here we are able to trace the changes of the brainwaves as they respond to the different conditions. Although the sample set is small, 14 out of 16 measurements were as expected. This is a clear indication that this biometric is a strong choice for further studies with larger sample sets.

Overall there is a mild increase in relaxation metrics such as cardiovascular and microcirculation scores, and there is not enough data to be conclusive. The sponsor hopes to model an excellent method for studying the effects of BB on the cardiovascular system, but a larger sample size is needed for any true narrative to be discovered.

As regards the circadian rhythms component of the experiment, there was no way to discern a difference between the morning and afternoon data, because they were experiencing different conditions. In fact, the design of the experience was such that everyone did the same thing at the same time for the express purpose that there be no difference in the data. Accounting for circadian rhythm is an important and suggested protocol for testing stimulation therapies, including BB (Webb and Dube, 1981; Rossi, 1986; Shannahoff-Khalsa, 1991; Atwater, 2001).

Given the research we would expect to see that the introverted subject is less affected than the extroverted subjects (Stelmack et al., 1993; Chaieb et al., 2015), and we have hypothesized a potential difference, but again, there is not enough data to come to a full conclusion. Either way the sponsor is wanting to model that this data should be included in every BB study given the research that introversion and extroversion are pertinent factors (Chaieb et al., 2015). There is a growing field of personality assessment and music preferences, indicating that these factors are of importance (Rentfrow et al., 2011; Anderson et al., 2021). A similar study with a greater number of participants is suggested.

## Conclusion

The aim of this case study is to assess the effects of adding BB to music and to brown noise on the brain and the physiology of four study participants who were seeking relief from stress and/or relaxation. As seen in the scalp topography maps and the cardiovascular data in particular, each participant had very different responses to the four auditory conditions, yet data from the brain activity of all four participants showed in visual and numerical representation that the effects of BB were deeply embedded within the mind. All four study participants experienced an improvement in brain function and had a calmer brain after adding BB to brown noise or to music plus brown noise. Most also showed an improvement in microcirculation or cardiovascular score after listening to music plus brown noise and BB. Since the BB were not audible, these effects could not be attributed to the placebo effect.

Listening to BB has been shown to induce synchronization of the brain hemispheres (Solcà et al., 2016) and BB help us to study or to effect cognitive brain function (Becher et al., 2015; Beauchene et al., 2016; Garcia-Argibay et al., 2019). When BB are used in a well- researched, conscious, evidence-based manner, there is a large amount of evidence showing that BB can be helpful in the categories of sleep (Gantt et al., 2017; Jirakittayakorn and Wongsawat, 2017; Bang et al., 2019; Lovati et al., 2019), anxiety (Isik et al., 2017; Garcia-Argibay et al., 2019), stress (Gantt et al., 2017), brain function (Gao et al., 2014; Reedijk et al., 2015), attention/cognition (Colzato et al., 2017; Lim et al., 2018; Garcia-Argibay et al., 2019; Axelsen et al., 2020), memory (Colzato et al., 2017; Gálvez et al., 2018; Lim et al., 2018); nervous system (da Silva et al., 2020), trance/meditation (Jirakittayakorn and Wongsawat, 2017; Perales et al., 2019), and as an analgesic (Garcia-Argibay et al., 2019; Perales et al., 2019; Gkolias et al., 2020). Gao et al. (2014) specifically suggested that treatments to improve clarity, focus, and sleep; to deepen meditation relaxation, to increase working memory and episodic memory; and to support ADHD may be the proper application of this tool. In addition, they discussed theta as a possible antidote for the Beta overwhelm that frequently occurs in patients with schizophrenia and depression (Krasnoff, 2021).

There are still many BB studies that show inconclusive or no effects (Solcà et al., 2016; López-Caballero and Escera, 2017; Lovati et al., 2019; Munro and Searchfield, 2019; Perez et al., 2020). This relatively new research field is still determining its parameters. Inconclusive studies could possibly be the result of selecting the wrong brainwave for the wrong task. Theta brainwaves are expected to induce relaxation, not increase focus, and we see Pluck and López-Águila (2019) conducted a controlled double blind experiment to explore the effect of theta (6 Hz) BB on cognitive fluency and fear. Beta brainwaves would have been a more expected choice for this study. In other studies, such as López-Caballero and Escera (2017), we see a usage time of 3 min for the BB, when research tells us that we need a minimum of 8 min (Garcia-Argibay et al., 2019). Inconclusive and negative result studies are essential for determining the parameters of this technology. What was once the main theory that BB operate through the process of entrainment, is now a discussion about stochastic resonance, brain regulation, the RAS, and neurotransmitters (Krasnoff, 2021). These are key research directions that will benefit the field of BB.

Lastly, it should be remembered that each auditory brain is different, representing the sum total of an individuals life experiences (Kraus, 2021). The auditory brain is a living system, teaching itself how to interpret sound and how to hear that sound with each afferent/efferent loop in the auditory pathway we call hearing (Kraus, 2021). Therefore, no two brains will respond the same to any auditory condition. What this means is that each individual must be individually diagnosed, assessed, and treated, when it comes to sound. A relaxation track for one individual might be a lullaby, and for another individual, a heavy metal group like Metallica. In this pilot study, we can see the brain activity respond differently to of all the auditory conditions, which underlines that individual response must be taken into account. Large sample sets help to offset these individual differences.

### Strengths

Strengths of this case study include consistency of time of data gathering. All testing was done at the same time of day for all participants to account for circadian rhythm. The BB were inaudible which eliminated placebo effect, and sequenced into the experiment such that we could immediately see whether there was a change or not. The study was double-blind and was conducted with two control conditions.

### Limitations

Limitations of the study included small number of participants–four. The small sample size may make it difficult to determine if a particular outcome is a true finding and limits generalization of the results to a larger population. There was a geographic limitation of the sample since all participants were from the same area near the research laboratory site. Since study participants were not confined to the laboratory during the duration of the study, external conditions could have influenced response to the auditory stimulation.

Highlights include: (1) The main aspects of BB in relation to the human brain and physiology, (2) Literature review of existing studies, and (3) BB as instrument for influencing brain function and supporting a calm state. These results are encouraging and warrant further research with more participants and different frequencies of BB and different musical tracks. Further study into the use of binaural beats for augmenting brainwaves can help diversify the knowledge about potential therapeutic uses. Investigations using large sample sets, different ranges of binaural beats, varying amounts of exposure time, a combination of multiple frequencies of beats, different carrier frequencies, and more sophisticated statistical analysis (Atwater, 2001; Huang and Charyton, 2008; Chaieb et al., 2015; Solcà et al., 2016; da Silva et al., 2020; Gkolias et al., 2020) are recommended to identify the most effective use of such strategies.

## Data availability statement

The original contributions presented in this study are included in the article/Supplementary material, further inquiries can be directed to the corresponding author.

## Ethics statement

Ethical review and approval was not required for the study on human participants in accordance with the local legislation and institutional requirements. The patients/participants provided their written informed consent to participate in this study. Written informed consent was obtained from the individual(s) for the publication of any potentially identifiable images or data included in this article.

## Author contributions

EK: writing, editing, supervision, auditory hardware, musical tracks, method, and conceptualization. GC: principal investigator. Both authors contributed to the article and approved the submitted version.
