# Supplementary material for: Case report: binaural beats music assessment experiment
Source: Front Hum Neurosci. 2023 May 5;17:1138650. doi: 10.3389/fnhum.2023.1138650 (PMC10196448; doi:10.3389/fnhum.2023.1138650)
Supplement: Supplementary file 2 [file Data_Sheet_2.docx]

**Pilot Study October 2021, Conditions 1-4**

Links for audio tracks

They are each present as a WAV and as an MP3.

Cut and paste this link into your browser

<https://www.dropbox.com/scl/fo/rdi8p9g8mwcwoho3z7387/h?dl=0&rlkey=pal3c1gu6duxashsccgdowpi9>
