# Supplementary material for: Case report: binaural beats music assessment experiment
Source: Front Hum Neurosci. 2023 May 5;17:1138650. doi: 10.3389/fnhum.2023.1138650 (PMC10196448; doi:10.3389/fnhum.2023.1138650)
Supplement: Supplementary file 3 [file Data_Sheet_3.docx]

**Big Five Trait Analysis - Sample**


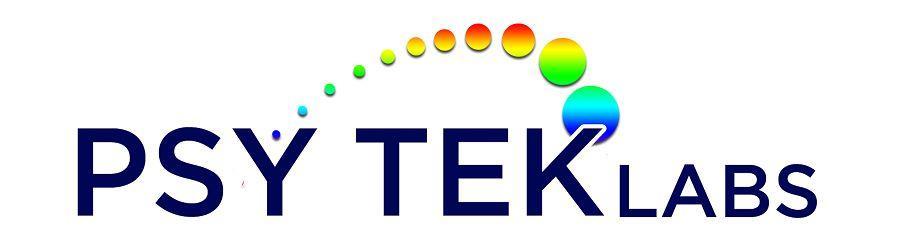


**Non-Invasive Scanning and Subtle Energy Testing Lab**

| item # | Extraversion Agreeableness Conscientiousness Neuroticism Openness | | | | |
| --- | --- | --- | --- | --- | --- |
| 1 | 3 | 3 | 5 | 1 | 5 |
| 2 | 4 | 5 | 5 | 2 | 5 |
| 3 | 5 | 3 | 5 | 4 | 4 |
| 4 | 4 | 5 | 5 | 3 | 5 |
| 5 | 3 | 5 | 5 | 2 | 5 |
| 6 | 5 | 2 | 5 | 2 | 5 |
| 7 | 3 | 5 | 5 | 2 | 5 |
| 8 | 3 | 5 | 5 | 2 | 5 |
| 9 |  | 5 | 4 |  | 5 |
| 10 |  |  |  |  | 5 |
| Total: | 30 | 38 | 44 | 18 | 49 |
| Ave: | 3.750 | 4.222 | 4.889 | 2.250 | 4.900 |
| SD: | 0.886 | 1.202 | 0.333 | 0.886 | 0.316 |

**Subject 2**

Male, 46. According to the table below, this subject shows a slight tendency to be an extravert and toward Consciousness, with strong tendencies toward Agreeableness, and Openness and low tendencies toward Neuroticism (quite stable emotionally).
