# Supplementary material for: Case report: binaural beats music assessment experiment
Source: Front Hum Neurosci. 2023 May 5;17:1138650. doi: 10.3389/fnhum.2023.1138650 (PMC10196448; doi:10.3389/fnhum.2023.1138650)
Supplement: Supplementary file 4 [file Data_Sheet_4.docx]

**PATIENT DEMOGRAPHIC DATA**

**Questions for Study Participants**

On a scale of 1-10 how stressed are you in general?

Does your stress affect your emotional health? Your physical health?

Do you have trouble sleeping?

To what do you attribute this stress in your life? i.e. work, relationships, money, parenting, emotions

What is the primary concern you would like to address with binaural beats? (to relax, to lower stress, regulate emotions better, sleep better)

What else have you tried? What has worked?

**Demographic Data**

Date of Birth

Gender

Race

Occupation

Cultural Background/Religion

Family Status: married, single, divorced, in a partnership

Previous Medical History- are you dealing with any significant health challenges (cancer, diabetes, chronic illness, heart illness, seizures)

mental health- are you dealing with any significant mental health challenges (anxiety, panic, depression, bipolar)

SUBJECT 1 is a 52-year-old-male (DOB 7/31/1969) dealing with high blood pressure and a stress level of 5 on a scale of 1 to 10. Subject 1 experiences stress due to money and career ambition, which affects his emotional health, and he is looking to binaural beats to regulate sleep and lower his stress levels. Exercise and meditation works for this individual.

SUBJECT is a 46-year-old male (DOB 9/9/72) dealing with high blood pressure and a stress level of 3 on a scale of 1 to 10. Subject 2 experiences little stress in his life, reports no physical, mental or sleep issues, and is looking to enjoy the retirement phase of his life. He is just beginning his journey into self-care.

SUBJECT 3 is a 33-year-old female (DOB 02/04/1988) who has a stress level of 5 on a scale of 1 to 10. Subject 3 experiences stress due to work and relationships, which affects her emotional and physical health. She would like to achieve more relaxation, lower her stress, and sleep better. Her self-care program involves relaxation music which helps her to fall asleep at night immediately. She reports no significant physical or mental issues and is taking no medications.

SUBJECT 4 is a 52-year-old female (DOB 3/22/1969) who has predominantly anxiety at a level of 3 on a scale of 1 to 10. Subject 4 experiences menopausal symptoms such as anxiety and poor sleep. Her anxiety is amplified from work and poor organization/planning ahead skills, and she would like to use binaural beats to experience greater relaxation. She has tried over the counter sleep aids.
