# Supplementary material for: Case report: binaural beats music assessment experiment
Source: Front Hum Neurosci. 2023 May 5;17:1138650. doi: 10.3389/fnhum.2023.1138650 (PMC10196448; doi:10.3389/fnhum.2023.1138650)
Supplement: Supplementary file 5 [file Data_Sheet_5.docx]

**Relaxation Script for Binaural Beats Case Study**

Take a moment to relax before you listen to this audio. Close your eyes and place your feet on the floor and your hands on your lap. Imagine a grounding cord connecting you from the base of your spine down to the center of the earth. Take three deep breaths to the count of four. One, two, three, four. Inhale and exhale. Two, three four. Inhale two, three, four and exhale two, three, four. Inhale two, three, four and exhale two, three, four. You are now relaxed. You may open your eyes and begin listening to the audio.
