# Supplementary material for: Case report: binaural beats music assessment experiment
Source: Front Hum Neurosci. 2023 May 5;17:1138650. doi: 10.3389/fnhum.2023.1138650 (PMC10196448; doi:10.3389/fnhum.2023.1138650)
Supplement: Supplementary file 6 [file Data_Sheet_6.docx]

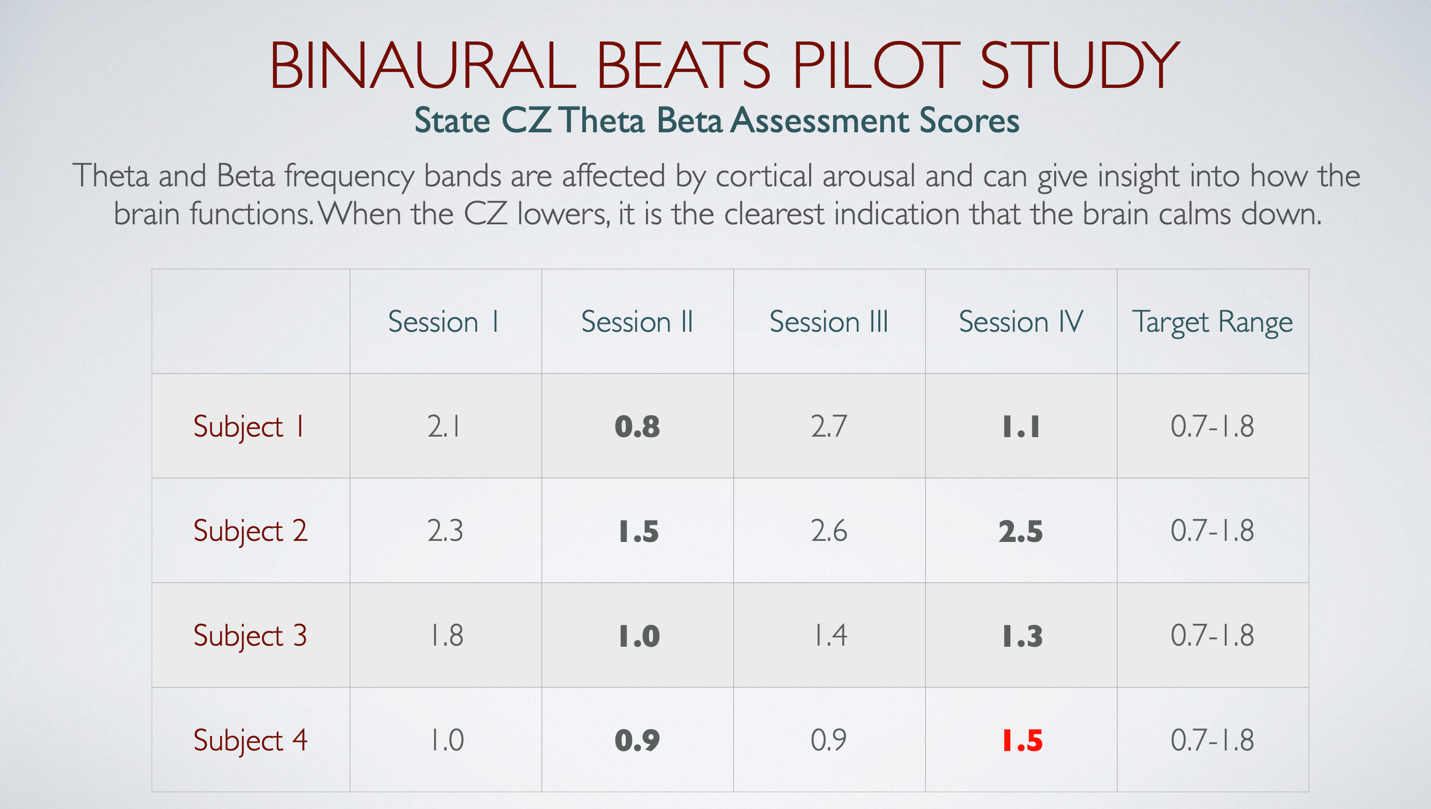


In session II, we added the binaural beats to the plain brown noise. In session IV, we added the binaural beats to the music track plus brown noise. In 7 conditions, we saw the expected lowering of numbers, indicating the brain had calmed down.

NOTE: The red color represents the one unexpected score. We expected the scores to lower when the participant relaxed, but in this one case that did not happen.
