# Supplementary material for: Case report: binaural beats music assessment experiment
Source: Front Hum Neurosci. 2023 May 5;17:1138650. doi: 10.3389/fnhum.2023.1138650 (PMC10196448; doi:10.3389/fnhum.2023.1138650)
Supplement: Supplementary file 7 [file Data_Sheet_7.docx]

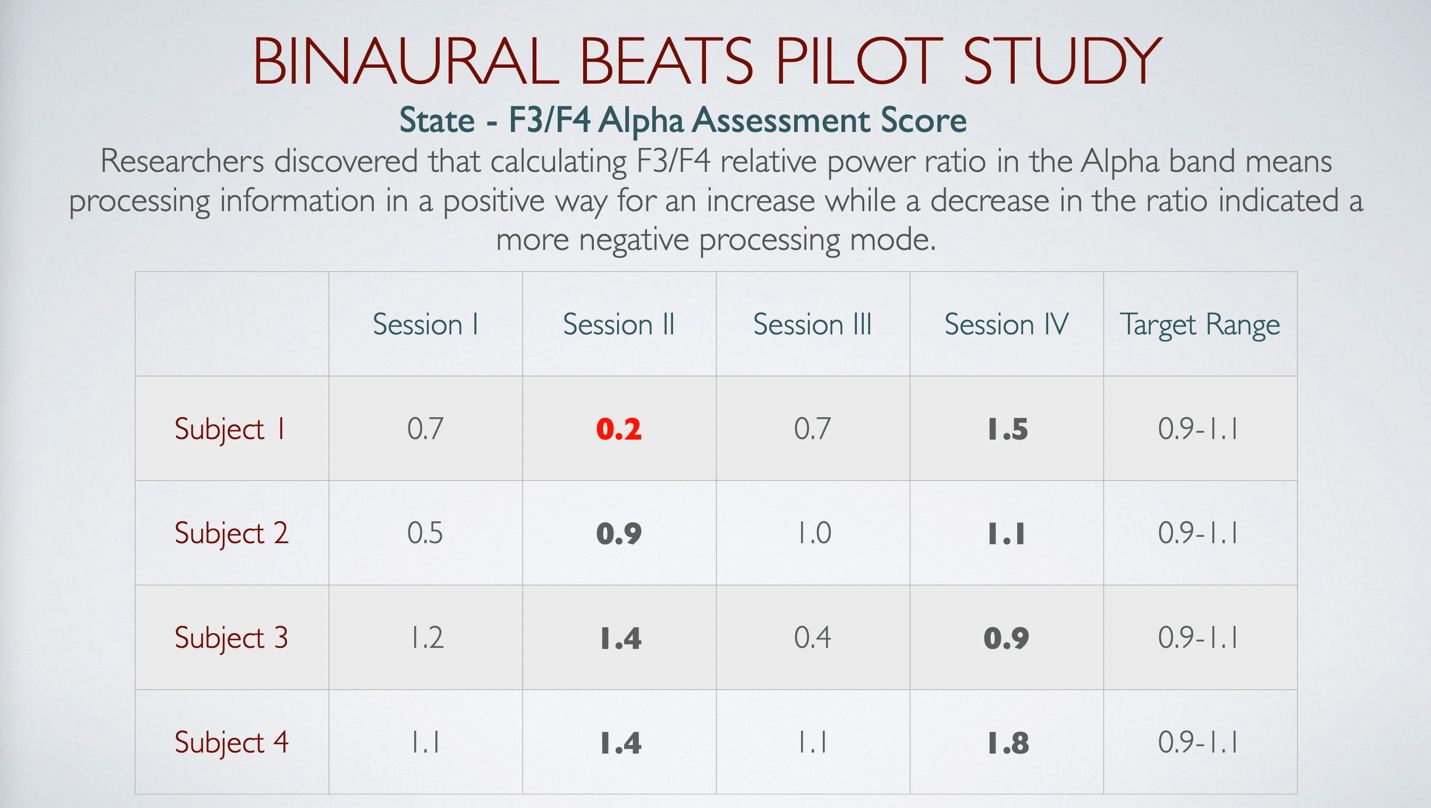


In session II, we added the binaural beats to the plain brown noise. In session IV, we added the binaural beats to the music track plus brown noise. In 7 conditions, we saw the expected increasing of numbers, indicating a more positive processing mode.

NOTE: The red color represents the one unexpected score. We expected the scores to raise when the participant relaxed and felt more positive, but in this one case that did not happen.
