# Supplementary material for: Case report: binaural beats music assessment experiment
Source: Front Hum Neurosci. 2023 May 5;17:1138650. doi: 10.3389/fnhum.2023.1138650 (PMC10196448; doi:10.3389/fnhum.2023.1138650)
Supplement: Supplementary file 8 [file Image_1.pdf]

Brief Mood Introspection Scale (BMIS)

Overall, my mood is:
Very Unpleasant Very Pleasant
-10 -9 -8 -7 -6 -5 -4 -3 -2 -1 0 1 2 3 4 5 6 7 8 9 10

**Brief Mood Introspection Scale (BMIS)**	**Session 1 BN**	**Session 2 BN + BB**	**Session 3 MUSIC + BN + BB**	**Session 4 MUSIC + BB**
**Subject 1**	4	**6**	7	**7**
**Subject 2**	0	**1**	*	*
**Subject 3**	-5	**4**	-1	**1**
**Subject 4**	8	**7**	8	**8**

BN = Brown Noise BB= Binaural Beats *= not obtained Red = unexpected data
