# Supplementary material for: Case report: binaural beats music assessment experiment
Source: Front Hum Neurosci. 2023 May 5;17:1138650. doi: 10.3389/fnhum.2023.1138650 (PMC10196448; doi:10.3389/fnhum.2023.1138650)
Supplement: Supplementary file 9 [file Image_2.pdf]

**MENLASCAN RESULTS SUBJECT 1**	**MICROCIRCULATION SCORE**	**CARDIOVASCULAR SCORE**
BASELINE	34	10
CONDITION 1<br>AFTER BROWN NOISE	20	**50**
**CONDITION 1 AFTER BN & BB**	**31**	**50**
BASELINE	33	18
CONDITION 2<br>AFTER MUSIC & BN	26	30
**CONDITION 2 AFTER MUSIC & BN & BB**	**46**	**28**
**MENLASCAN RESULTS SUBJECT 2**	**MICROCIRCULATION SCORE**	**CARDIOVASCULAR SCORE**
BASELINE	54	95
CONDITION 1<br>AFTER BROWN NOISE	52	95
**CONDITION 1 AFTER BN & BB**	**47**	**75**
BASELINE	63	95
CONDITION 2<br>AFTER MUSIC & BN	54	85

**CONDITION 2 AFTER MUSIC & BN & BB**	**58**	**95**
**MENLASCAN RESULTS SUBJECT 3**	**MICROCIRCULATION SCORE**	**CARDIOVASCULAR SCORE**
BASELINE	34	10
CONDITION 1<br>AFTER BROWN NOISE	20	50
**CONDITION 2 AFTER BN & BB**	**31**	**50**
BASELINE	74	73
CONDITION 3<br>AFTER MUSIC & BN	62	71
**CONDITION 4**<br>AFTER MUSIC & BN & BB	**20**	**83**
**MENLASCAN RESULTS SUBJECT 4**	**MICROCIRCULATION SCORE**	**CARDIOVASCULAR SCORE**
BASELINE	40	75
CONDITION 1<br>AFTER BROWN NOISE	41	65
**CONDITION 2**<br>**AFTER BN & BB**	**35**	**81**
BASELINE	43	83

CONDITION 3<br>AFTER MUSIC & BN	37	73
**CONDITION 4**<br>**AFTER MUSIC & BN & BB**	**40**	**85**

NOTE: All Binaural Beats results are highlighted in **bold.** We are expecting all results to increase showing increased bloodflow and relaxation. Unexpected results that lower instead of raise are highlighted in red.

The Menlascan results provided us with measurements on the cardiovascular system, which refers to the heart (cardio) and blood vessels (vascular). This system distributes blood to all parts of the body, and is governed by the autonomic nervous system (ANS). The ANS is a component of the peripheral nervous system that regulates involuntary physiologic processes including heart rate, blood pressure, respiration, digestion, and sexual arousal (https://www.ncbi.nlm.nih.gov/books/NBK539845/). Microcirculation is blood flow through the smallest vessels of the cardiovascular system. The most important results here are the improvement of cardiovascular and microcirculation scores as the ANS is prone to rapid changes with emotions.

Subject 1 had the highest microcirculation score with music + BB on day 1, and on day 2 an increased cardiovascular score from music + BB (although just music increased cardiovascular as well). In subject 2, day 1 shows no improvement with adding the BB. On day 2, Music + BB increase the cardiovascular and microcirculation score. In Subject 3, our introverted subject, we see the least effect, with BN and BN + BB increasing only cardiovascular score on day 1, and on day 2, music + BB provide a mild cardiovascular improvement only. In subject 4, there is a mild improvement only in cardiovascular when adding BB, and on day 2, has a mild improvement in cardiovascular. There is a mild increase in relaxation metrics such as cardiovascular and microcirculation scores, however there is not enough data to be conclusive. The sponsor hopes to model an excellent method for studying the effects of binaural beats on the cardiovascular system, but a larger sample size is needed for any true narrative to be discovered.
